# Effects of single session transcranial direct current stimulation on aerobic performance and one arm pull-down explosive force of professional rock climbers

**DOI:** 10.3389/fphys.2023.1153900

**Published:** 2023-04-06

**Authors:** Jia Luo, Caihua Fang, Sen Huang, Jinlong Wu, Bowen Liu, Jingxuan Yu, Wen Xiao, Zhanbing Ren

**Affiliations:** ^1^ Key Laboratory of Kinesiology Evaluation and Recovery of General Administration of Sport of China, Sports Science Institute of Hunan, Changsha, China; ^2^ College of Physical Education, Southwest University, Chongqing, China; ^3^ College of Physical Education, Shenzhen University, Shenzhen, China

**Keywords:** transcranial direct current stimulation, rock climbing, athletes, sports performance, aerobic performance, explosive force

## Abstract

**Objective:** To explore the effects of single-session transcranial direct current stimulation (tDCS) on aerobic performance and explosive force in the one-arm pull-down of long-term trained rock climbers.

**Method:** Twenty athletes (twelve male and eight female) from the Rock Climbing Team of Hunan province (Hunan, China) were selected for a randomized double-blind crossover study. After baseline tests, All subjects visited laboratories twice to randomly receive either sham or a-tDCS at a current intensity of 2 mA for 20 min. The two visits were more than 72 h apart. Immediately after each stimulation, subjects completed a 9-min 3-level-load aerobic test and a one-arm pull-down test.

**Results:** Differences in the heart rate immediately after 9-min incremental aerobic exercises revealed no statistical significance between each group (*p* > 0.05). However, the decrease in heart rate per unit time after exercise after real stimulation was significantly better than before stimulation (*p* < 0.05), and no statistical significance was observed between after sham stimulation and before stimulation (*p* > 0.05). One-arm pull-down explosive force on both sides after real stimulation was improved by a-tDCS compared with before stimulation, but with no significant difference (*p* > 0.05). Real stimulation was significantly improved, compared with sham stimulation on the right side (*p* < 0.05).

**Conclusion:** Single-session tDCS could potentially benefit sports performance in professional athletes.

## 1 Introduction

As a new non-invasive nerve regulation technology, transcranial direct current stimulation (tDCS) applies a weak direct current (1–2 mA) on the scalp in the form of electrodes, lasting for 5–20 min (Nitsche et al., 2008). Its earliest introduction is for the treatment of clinical diseases, mainly involving psychiatric diseases such as pain, Parkinson’s disease, stroke, Alzheimer’s disease, depression, schizophrenia, and craving/addiction, and it has proved a significant therapeutic effect (Lefaucheur et al., 2017). Some studies have shown that tDCS can regulate the subthreshold of neuron membrane potential, changing cortex excitability and activity according to the direction of current passing through the target neuron (Purpura and McMurtry, 1965). In addition, it may also relate to neurotransmitter variation caused by current changes, effects of glial cells and microvessels, and inflammatory process regulation (Woods et al., 2016). However, the effects of tDCS depend on current intensity, polarity, relative position of electrode and intervention duration (Paulus, 2011; Angius et al., 2019a; Borges et al., 2020). Marom et al. (Bikson et al., 2016) have summarized more than 33,200 courses of treatment and 1,000 repeated courses of treatment on human subjects and discovered no reports of serious adverse effects or irreversible injuries. With advantages of safety, low cost, portability and ease of operation, this technology enjoys a broad application prospect in the field of brain science. In recent years, numerous studies have revealed that tDCS can help improve sports performance, including enhancement in motor learning (Debarnot et al., 2019), cognitive execution (Yu et al., 2018), muscle strength (Alix-Fages et al., 2020; Kenville et al., 2020), muscle endurance and fatigue perception (Park et al., 2010; Angius et al., 2019b). Due to its potentiality in improving the above sports performance, the current research on the potential application of tDCS in sports related skills has come to the foreground. So far, the research on the neurophysiological mechanism underlying tDCS’s regulation of motor performance is relatively weak. It has been speculated that possible mechanisms of tDCS improving motor performance include: 1) increasing or decreasing the resting membrane potential leads to an increase or decrease of nerve excitability (Gandiga et al., 2006); 2) Regulating and altering synaptic activity (Stagg and Nitsche, 2011); 3) Improving functional connectivity of various brain regions (Meinzer et al., 2012).

An exploration of safe and effective improvement of athletes’ competitive level and performance is a core part in science and technology’ support in competitive sports. The neural plasticity of the human brain is that the neural circuit can be affected by external or internal factors and possess features of reorganization and reconstruction. Also, both disease and stress can cause changes in the synaptic function of the brain (Steinberg et al., 2018). As a stress event, sports training has a great impact on the structure and function of human brain. Research indicates that after long-term sports training, professional athletes are significantly different from ordinary people in brain structure, neural activation, fine regulation and other aspects. Transcranial direct current stimulation (tDCS) may further improve training results by regulating the brain regions that display the training-induced neural plasticity (Seidel-Marzi and Ragert, 2020). However, there are still controversies on the research results of professional athletes. Some studies have discovered that the utilization of tDCS has no (Valenzuela et al., 2019; Mesquita et al., 2020) or even deteriorating (Mesquita et al., 2019) effect on sports performance after an intervention on professional athletes. Other studies have proved that tDCS can significantly improve athletes’ performance (Kamali et al., 2019; Machado et al., 2019). At present, the relevant research is still rare, and causes for the different results are to be further studied. More evidence is needed to see whether tDCS can help to further improve the exercise ability at a high level of exercise.

Currently, most intervention methods used in scientific studies are single acute stimulation. Some results show that single acute tDCS stimulation can improve the body’s performance, such as muscle strength and endurance. Xiao (Xiao et al., 2020) et al. studied acute effects of a single high definition transcranial direct current stimulation (HD-tDCS) on foot muscle strength and static balance, and found that it has improved the toe flexor strength and static standing balance performance. Angius (Angius et al., 2019b)’s acute tDCS stimulation program enhanced the individual’s inhibitory control and endurance cycle performance. Halo Sport headset is a commercial device based on tDCS technology. Huang et al. (Huang et al., 2019) applied Halo Sport to the motor cortex of healthy adult men and found that it has a significant promoting effect on cycling power output and cognitive executive function. Currently, few studies have explored the effects of tDCS on professional rock climbers. Hence, this experiment takes professional rock climbers as subjects and uses Halo Sport transcranial DC headset to observe the effect of an acute intervention on their aerobic performance and one-arm pull-down explosive force, so as to further enrich the experimental research in this field.

## 2 Methods

### 2.1 Subjects

Twenty athletes (twelve male and eight female) from the Rock Climbing Team of Hunan province (Hunan, China) were chosen for this study. Subjects were recruited from filling questionnaires with basic information like age, years of training, etc., Their ages were 17.11 ± 2.38 years old, with a training duration of 5.80 ± 2.78 years, height of 166.82 ± 7.62 cm and weight of 58.18 ± 7.82 kg. The inclusion criteria are as follows: 1) between 15 and 20 years old; 2) healthy with normal muscle function; 3) at least 3 years of rock climbing training; 4) all athletes are from the same team to ensure that the training time and frequency are exactly the same. The exclusion criteria are as follows: 1) athletes with poor sleep, drinking or coffee habits, and chronic mental stress. 2) with a history of head injuries. Participants who fell into the exclusion criteria were excluded. Health conditions of participants were self-reported. The laboratory environment was quiet and stable during treatment (temperature 22° ± 0.5°C, humidity 47% ± 4%).

All subjects provided informed consent prior to the experiment, and were aware of the test content and experimental process prior to treatment. This research was approved by the Hunan Institute of Sports Science Committee (Agreement No. 2022112201).

### 2.2 Experimental procedure

We utilized randomized, double-blind and crossover experiments, Written informed consent was obtained from all participants. Participants were asked to wear sports clothes during test. Two or three simulation tests were conducted before formal testing to help athletes familiarize themselves with the test methods. 3 min warm-up exercise before formal test. Each subject received three tests, namely, a baseline test, a real stimulation, and a sham stimulation, and the interval between two tests was 72 h. All of the tests were conducted in the morning. In the formal experiments, the first time was the baseline test of motor ability, include Aerobic Performance Test and One-arm Pull-down Explosive Force Test, the second and third assessments were the true or sham stimulation, each with a motor ability test afterwards.

**FIGURE 1:**
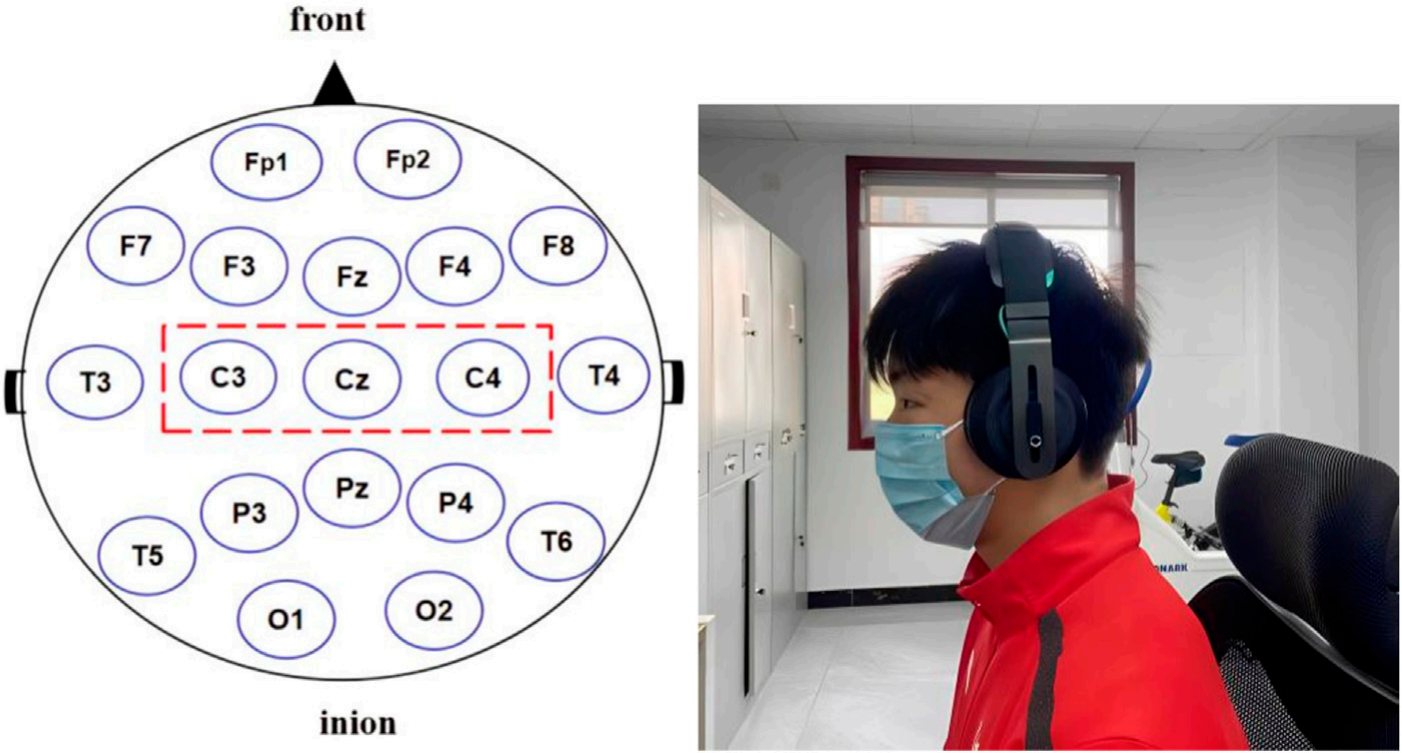
The red dotted box represents the stimulated areas in the picture on the left. The image on the right is an athlete undergoing treatment.

**FIGURE 2:**
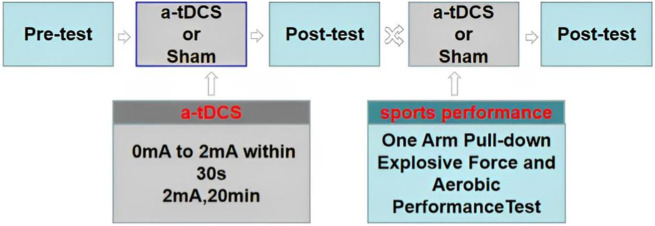
Study design. Pre-test and post-test indicate sports performance test, a-tDCS represents current gradually increasing from 0 mA to 2 mA within 30 s, and the whole test lasted for 20 min.

These two test procedures were the same as the first baseline test. During the intervention experiment, the subjects sat in a chair and wore Halo Sport transcranial DC earphones (Halo Neuroscience, United States). The current-stimulated portions of the brain were the left and right primary motor cortex, in accordance with the 10–20 international electrode positioning system. The Halo Sport was placed on the subjects’ head after the device’s integrated electrodes were saturated with water. The associated mobile application was used to confirm a strong connection. In the real stimulation group, the current gradually increased from 0 mA to 2 mA within 30 s, and the whole testing lasted for 20 min; in the sham stimulation-controlled group, the current gradually increased from 0 mA to 2 mA within 30 s, then dropped to 0 mA within 30 s, and subjects continued to wear their headphone until 20 min of testing concluded. In testing, only one athlete and one tester were left in the quiet laboratory. In the real stimulation testing, some athletes felt itchy and slight tingling on the head, with no other adverse reactions (Figure 1 and Figure 2).

### 2.3 Aerobic performance test

A Monark powered bicycle (Monark 928E, Sweden) was used for aerobic tests. The experimental methods refer to Li et al. (Li et al., 2010), Three minute incremental exercise can be an effective way to test aerobic capacity. The subjects wore a Polar heart rate strap and received three-level load exercise tests of 50 w, 100 w, and 150 w, respectively. The exercise time of each level of load was 3 min, meaning 9 min in total. Heart rates at all three levels of load during exercise, immediately after exercise, and the first, third, and fifth minutes of the recovery period after exercise were recorded. The recovery heart rate per unit time was then calculated. This was plotted using the following equation: recovery heart rate per unit time (10 s) = (heart rate in the fifth minute of recovery period—heart rate immediately after the exercise)/30.

### 2.4 One-arm pull-down explosive force test

Keiser Pneumatic Resistance Training Machines (Keiser3025, United States) was used for this test. those machines seem credible for 1RM testing (Lu et al., 2021). During the first time period, the best resistance test was conducted. The subject grasped the pull strap with both hands, and first took a light resistance test three times, and then the heavy resistance test for three times. This way, the best resistance force that could produce the maximum explosive force was calculated. With this resistance value, a full standardized one-arm high pull-down test was conducted three times, and the interval between two tests was 1 min. The optimal value was then taken.

### 2.5 Data analysis

SPSS 22.0 was used data for statistical analysis. The data, expressed as the mean ± standard deviation (x ± s), were assessed by two-way ANOVA (stimulus mode × time). Stimulus modes of real stimulation and sham stimulation and test time points were used as independent variables, while the test results were used as dependent variables. Mauchly’s test was used to test the sphericity hypothesis. When the sphericity test was met, a paired sample t-test was used to compare each group, and when it was not met, multivariate tests were used. LSD was utilized for pairwise comparisons to analyze the differences of indicators at different times and in different intervention modes. Cohen’s d was used to express the effect size: 0.2 was a weak effect, 0.5 was a medium effect, and 0.8 was a strong effect. *p* < 0.05 indicated that a difference was significant.

## 3 Results

### 3.1 Aerobic performance test

**FIGURE 3:**
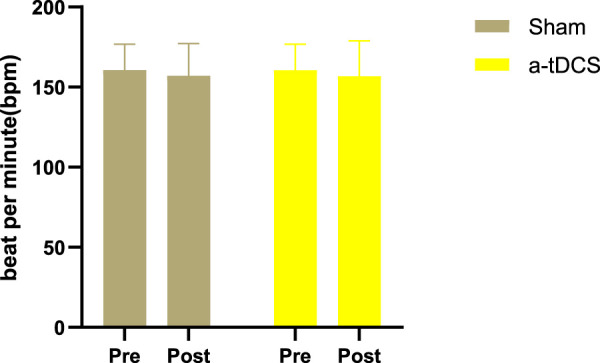
Immediate heart rate after 9-min aerobic activity. The white bars represent real stimulation compared to pre-stimulation, and the gray bars represent sham stimulation compared to pre-stimulation. “Pre” means before stimulation, and “post” means after stimulation.

Figure 3 displays the immediate heart rate after 9 min of aerobic activity. There was no interaction between stimulation and test time (F = 1.254, *p* = 0.298). T-test results for paired samples were used to assess the immediate heart rate after three-level load exercise, and no significant difference was observed between after real stimulation and before stimulation (*p* = 0.281, Cohen’s d = 0.248), after sham stimulation and before stimulation (*p* = 0.278, Cohen’s d = 0.249), or after real stimulation and after sham stimulation (*p* = 0.882, Cohen’s d = 0.034). these results all show small effect sizes.

**FIGURE 4:**
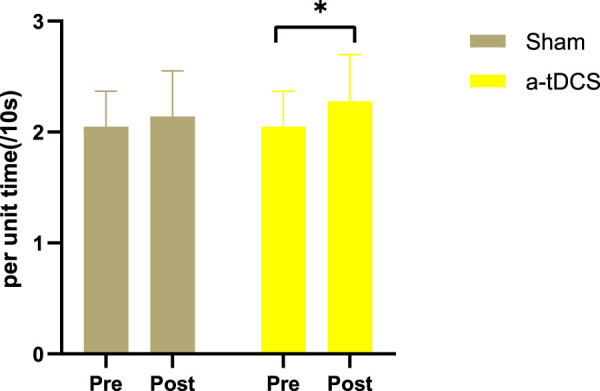
Drop in heart rate per unit time. The white bars represent real stimulation compared to pre-stimulation, and the gray bars represent sham stimulation compared to pre-stimulation. “Pre” means before stimulation, and “post” means after stimulation. Note: * indicates *p* < 0.05.

Figure 4 shows the drop rate in heart rate per unit time. There was no statistically significant interaction between stimulation and test time (F = 2.441, *p* = 0.101). The paired sample t-test results showed that after real stimulation was significantly higher than before stimulation (*p* = 0.045, Cohen’s d = 0.480), and slightly higher than after sham stimulation with no significant difference (*p* = 0.443, Cohen’s d = 0.175). After sham stimulation also showed no statistical difference compared with before stimulation (*p* = 0.151, Cohen’s d = 0.335). these results all show small effect sizes.

### 3.2 One-arm pull-down explosive force test

**FIGURE 5:**
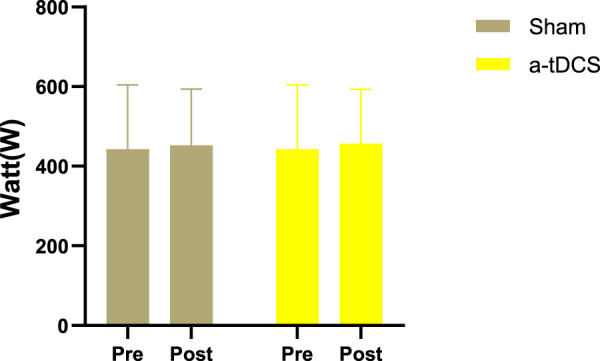
Left-arm pull-down explosive force. The white bars represent real stimulation compared to pre-stimulation, and the gray bars represent sham stimulation compared to pre-stimulation. “Pre” indicates before stimulation, while “post” represents after stimulation.

**FIGURE 6:**
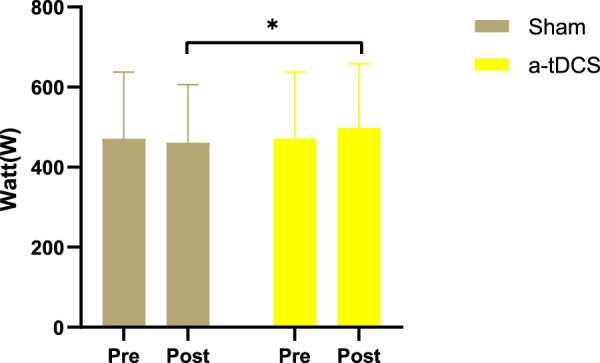
Right-arm pull-down explosive force. The white bars represent real stimulation compared to pre-stimulation, while the gray bars represent sham stimulation compared to pre-stimulation. “Pre” means before stimulation, and “post” means after stimulation. Note: * means *p* < 0.05.

Figures 5, 6 show that there was no interaction between the stimulation of left-arm pull-down explosive force and test time (F = 0.796, *p* = 0.459). No significant difference was detected between after real stimulation and before stimulation (*p* = 0.334, Cohen’s d = 0.222). After sham stimulation improved slightly compared with before stimulation, but there was no statistically significant difference (*p* = 0.443, Cohen’s d = 0.175). No significant difference was observed between the real and sham stimulation groups (*p* = 0.736, Cohen’s d = 0.076). The right-arm pull-down explosive force stimulation showed no interaction with test time (F = 0.417, *p* = 0.662). Compared with before stimulation, the after real stimulation group was improved but without a significant difference (*p* = 0.279, Cohen’s d = 0.249), and after real stimulation was significantly improved compared with after sham stimulation (*p* = 0.046, Cohen’s d = 0.165), and there was no significant difference between after sham stimulation and before stimulation (*p* = 0.469, Cohen’s d = 0.478). these results all show small effect sizes.

## 4 Discussion

Rock climbing has attracted much international attention after its inclusion as an official event in the next Olympic Games, and thus, improvement of its scientific training level has become a focus in sports research. Currently, tDCS, a new technology to assist athletic sports, is at the forefront of sports training, but reports on its application in professional athletes are rare and inconsistent. Thus, we sought to explore the effects of tDCS on the athletic ability of long-term professionally trained rock-climbing athletes. To this end, athletes’ bilateral motor cortexes were stimulated using Halo Sport transcranial DC earphones at an intensity of 2 mA for 20 min. The experimental results showed that acute tDCS stimulation had no distinct effect on the heart rate immediately after intensity-incremental aerobic load testing. However, it did significantly improve the heart rate decline during the recovery period after aerobic exercise, and a single acute stimulation impacted the explosive force in one-arm pull-down tests of rock climbers.

### 4.1 Effects of tDCS on aerobic performance in rock climbers

Aerobic capacity is one of the key factors affecting the performance of elite rock climbers, improving whole-body aerobic capacity is a notable contribution to Rock climbing performance (Fryer et al., 2018). Research showed that a steady-heart rate or entrance into a stable state was observable approximately 3 min after moderate intensity uniform motion, and the heart rate at this moment reflects aerobic exercise intensity (Du et al., 1998). Therefore, this study adopted an aerobic exercise program with three level incremental loads, and each level lasted for 3 min. During true and sham stimulations, the heart rates immediately after exercise were slightly improved compared with before stimulation, but this was not statistically significant. This result was consistent with current results at home and abroad. Some research (Park et al., 2010; Holgado et al., 2019) has shown that tDCS has no effect on cardiorespiratory responses during exercise, such as heart rate (HR) and oxygen uptake (VO2). For example, Mesquita et al. (Mesquita et al., 2020) studied whether anode tDCS had a significant effect on aerobic performance in professional taekwondo athletes. Machado et al. (Da Silva Machado et al., 2021) also reported that the heart rate of 80% cycle endurance tests and other physiological indices of endurance athletes did not change after 20-min high-precision tDCS (HD tDCS) (2.4 mA) and traditional tDCS (2.0 mA). The reason may have been that the communication between the central nervous system and the exercise unit was only regulated by afferent responses or there was a ceiling effect. Previous studies have also revealed (Hilz et al., 2002; Tanaka et al., 2009) that the heart rate during exercise is controlled by the autonomic nervous system, thus increasing or decreasing parasympathetic and sympathetic functions can affect fatigue and sports performance. Meta analysis has shown that non-invasive brain stimulation (NIBS), including tDCS, can affect cardiovascular and autonomic nervous system activities (Makovac et al., 2017). Okano et al. (Okano et al., 2015) applied tDCS to the temporal lobe cortex (TC) of professional cyclists, using a 2-mA current for 20 min. They observed that the heart rate decreased under sub-maximum workload, suggesting that anode tDCS seemed to induce cardiac autonomic control and the improvement of cardiac efficiency during aerobic exercise. Another study (Kamali et al., 2019) also reported a decrease of heart rate of 4.9% in bodybuilders when they were doing 12 knee joint stretching exercises 13 min after a 2-mA current stimulation of the M1 and left TC area. However, the current evidence is still inconclusive about the effects of tDCS on heart rate during exercise, and no significant effect was observed in this study. It has been speculated that results can affected by factors like the setting of each exercise program, electrode stimulation parameters and brain positioning, and any slight improvement of aerobic capacity on athletes in this experiment may be attributable to some positive psychological implications of the intervention mode.

Our study highlighted that tDCS can promote the recovery of heart rate after exercise. Moreira et al. (Moreira et al., 2021) stimulated the left and right dorsolateral prefrontal cortex (F3 and F4) of professional football players with 2 mA for 20 min to test the heart rate recovery 1 min after exercise. They also found that tDCS had a significant effect on the rapid recovery of early heart rate. They suggested that tDCS intervention might lead to changes in brain regions related to autonomous control, and ultimately caused the activation of parasympathetic autonomic activities to optimize the recovery of players. In addition, Montenegro et al. (Montenegro et al., 2013), after a research on 11 healthy men, discovered the regulatory effect of tDCS on physiological function after exercise. They speculated that the effect of tDCS on the prefrontal cortex may have a beneficial impact on autonomic respiratory control by increasing VO2 and energy consumption after aerobic exercise. Although there is scant relevant research, the effect of tDCS on regulating autonomic nervous activity and promoting the recovery of athletes after sports is an important contribution to this field.

### 4.2 Influence of tDCS on the upper limb explosive force of rock climbers

Strong back muscle strength is crucial to rock-climbers. As a classic action in physical training, high pull-down tests reflects the muscle strength of the upper back, latissimus dorsi and trapezius. tDCS appeared to result in better enhancement of muscle strength than muscle endurance and other sports performance metrics, and this enhancement was consistent across different subjects, such as ordinary subjects and patients (Sun et al., 2021; Chinzara et al., 2022). Patel et al. (Patel et al., 2019) summarized reports on the effects of tDCS on the upper limb motor performance in healthy adults, and found that tDCS could significantly increase upper limb strength. Hazime et al. (Hazime et al., 2017) conducted tDCS on female handball players, participating in regional and national competitions, and discovered that it could increase their maximum isometric contraction strength of the internal and external rotation shoulder muscles. Lattari et al. (Lattari et al., 2016) carried out a tDCS program on bodybuilders who had received resistive exercise training for at least 3 months and noted increased strength in their elbow flexion. The results of this study also showed that tDCS applied to the primary motor cortex may enhance the explosive force of one-arm pull-down in rock climbers. The neurophysiological mechanism of tDCS’s improvement of motor performance has been reported in a small number of reports. Hendy et al. (Hendy et al., 2014) found that the application of a 20-min current of 2 mA to the right motor cortex could significantly improve maximum autonomic strength of the untrained wrist, and along with corticospinal excitability, reduced inhibition in the short interval cortex and increased cross activation. In addition, Alix et al. (Alix-Fages et al., 2020) conducted a 15-min 2 mA tDCS stimulation of the dorsolateral prefrontal cortex in 14 healthy men, and discovered that with one repeat maximum, the force-velocity relationship parameters were not improved. This was because other factors such as effect size, stimulation parameters, genetics, gender, experience and even skull thickness may regulate tDCS effects (Zimerman and Hummel, 2010; Craig et al., 2020; Hunold et al., 2021).

There were some limitations in this study that need to be noted, such as the major limitation of the present study, which was that the sample size was too small In order to avoid sample differences caused by a large age span, the sample size needs to be further expanded in future studies.

## 5 Conclusion

tDCS as a new technology to assist athletic sports could potentially benefit the sports performance of professional athletes. Future studies should be conducted with larger samples to increase the statistical power of these findings.

## Data Availability

The raw data supporting the conclusion of this article will be made available by the authors, without undue reservation.
